# MeLSI: Metric Learning for Statistical Inference in microbiome community composition analysis

**DOI:** 10.1128/msystems.00407-26

**Published:** 2026-06-29

**Authors:** Nathan Bresette, Aaron C. Ericsson, Carter Woods, Ai-Ling Lin

**Affiliations:** 1 Roy Blunt NextGen Precision Health, University of Missouri 14716, Columbia, Missouri, USA; 2 Institute for Data Science and Informatics, University of Missouri 14716, Columbia, Missouri, USA; 3 University of Missouri Metagenomics Center, Columbia, Missouri, USA; 4 Department of Pathobiology and Integrative Biomedical Sciences, University of Missouri, Columbia, Missouri, USA; 5 Department of Radiology, University of Missouri, Columbia, Missouri, USA; 6 Division of Biological Sciences, University of Missouri 14716, Columbia, Missouri, USA; Drexel University, Philadelphia, Pennsylvania, USA; The University of Hong Kong, Hong Kong, Hong Kong

**Keywords:** microbiome analysis, metric learning, beta diversity, community composition, PERMANOVA, distance metrics, permutation testing

## Abstract

Microbiome beta diversity analysis relies on distance-based methods, including permutational multivariate analysis of variance (PERMANOVA) combined with fixed ecological distance metrics (Bray-Curtis, Euclidean, Jaccard, and UniFrac), which treat all microbial taxa uniformly, regardless of their biological relevance to community differences. This “one-size-fits-all” approach may miss subtle but biologically meaningful patterns in complex microbiome data. We present Metric Learning for Statistical Inference (MeLSI), a novel machine learning framework that learns data-adaptive distance metrics optimized for detecting community composition differences in multivariate microbiome analyses. MeLSI employs an ensemble of weak learners using bootstrap sampling, feature subsampling, and gradient-based optimization to learn optimal feature weights, combined with rigorous permutation testing for statistical inference. The learned metrics can be used with PERMANOVA for hypothesis testing and with principal coordinates analysis for ordination visualization. Comprehensive validation on synthetic benchmarks and real data sets shows that MeLSI maintains proper type I error control while delivering competitive or superior statistical power for detecting subtle community shifts and, crucially, supplies interpretable feature-weight profiles that clarify which taxa drive group separation. On the DietSwap data set, MeLSI was the only method to achieve significance at α = 0.05, demonstrating that adaptive weighting can detect diet-induced community shifts that fixed metrics miss. Across all data sets, the learned feature weights identified biologically relevant taxa while providing actionable insight that no fixed distance metric can supply. MeLSI therefore offers a statistically rigorous tool that augments beta diversity analysis with transparent, data-driven interpretability.

**IMPORTANCE:**

Understanding which microbes differ between groups of interest could reveal therapeutic targets and diagnostic biomarkers. However, current analysis methods treat all microbes equally (similar to using the same ruler to measure everything, regardless of what matters most). This means subtle but biologically important differences may go undetected, especially when only a few key species drive disease states while hundreds of “bystander” species add noise. Metric Learning for Statistical Inference (MeLSI) solves this by learning which microbes matter most for each specific comparison. In comparing male and female gut microbiomes, MeLSI identified specific bacterial families driving the differences, providing actionable biological insights that standard methods miss. This capability is particularly crucial for detecting early disease biomarkers, where differences are subtle and masked by biological variability. By telling researchers not just whether groups differ, but which specific microbes drive those differences, MeLSI accelerates the path from microbiome data to testable biological hypotheses and clinical applications.

## INTRODUCTION

### The microbiome and human health

The human microbiome, the complex community of microorganisms inhabiting our bodies, plays fundamental roles in health and disease (1, 2). Recent advances in high-throughput sequencing technologies have enabled comprehensive profiling of microbial communities, revealing associations between microbiome composition and diverse conditions, including inflammatory bowel disease, obesity, diabetes, and neurological disorders (3, 4). A central objective in microbiome research is comparing overall microbial community composition between groups of interest, typically assessed through beta diversity analysis, which studies compositional differences between samples.

### Current approaches and their limitations

Microbiome beta diversity analysis predominantly relies on distance-based multivariate methods, including permutational multivariate analysis of variance (PERMANOVA) combined with fixed ecological distance metrics (5, 6). Commonly used metrics include Bray-Curtis dissimilarity, Euclidean distance, Jaccard index, and phylogenetically informed metrics, including UniFrac (7). These approaches have proven valuable for hypothesis testing about community differences and visualization through ordination methods such as principal coordinates analysis (PCoA) (8).

However, fixed distance metrics suffer from a fundamental limitation. They apply the same mathematical formula to all data sets, treating all microbial taxa with equal importance regardless of their biological relevance to the specific research question (9). For instance, Bray-Curtis dissimilarity equally weights all taxa based on their relative abundances, while Euclidean distance treats all features identically. This “one-size-fits-all” approach may fail to capture subtle but biologically meaningful differences when only a subset of taxa drive group separation (10).

Furthermore, microbiome data present unique analytical challenges, including high dimensionality (often hundreds to thousands of taxa), compositionality (relative abundances sum to a constant), sparsity (many zero counts), and heterogeneous biological signal across features (11). Fixed metrics cannot adapt to these complexities in a data-driven manner.

### The need for statistical rigor

A critical requirement for any beta diversity method is proper statistical inference with controlled type I error rates (false positive rates). While machine learning approaches often prioritize predictive accuracy, hypothesis testing for community composition differences requires rigorous *F*-statistic and *P*-value calculation under the null hypothesis of no group differences (12). Permutation testing provides a non-parametric framework for valid inference that makes minimal distributional assumptions (13), making it particularly suitable for complex microbiome data and distance-based analyses such as PERMANOVA.

### Metric learning: an emerging paradigm

Metric learning, a branch of machine learning, offers a principled approach to address these limitations (14, 15). Rather than using fixed distance formulas, metric learning algorithms learn optimal distance metrics from data by identifying which features contribute most to separating groups of interest. In the context of supervised learning, metric learning algorithms optimize distance functions to maximize between-group distances while minimizing within-group distances (16, 17).

We formalize metric learning as follows: let $X\in R^{n\times p}$ denote a feature abundance matrix with *n* samples and *p* taxa, and let $y=\left(y_{1},\ldots ,y_{n}\right)$ denote group labels. A distance metric is parameterized by a positive semi-definite matrix $M\in R^{p\times p}$, where the Mahalanobis distance between samples *i* and *j* is $d_{M}\left(x_{i},x_{j}\right)=\sqrt{{\left(x_{i}-x_{j}\right)}^{T}M\left(x_{i}-x_{j}\right)}$. For diagonal M, this reduces to the weighted Euclidean distance with feature-specific weights *M_jj_* representing the learned contribution of feature *j* to the distance.

Mahalanobis distance learning (18) learns a positive semi-definite matrix M that defines distances as $d\left(x_{i},x_{j}\right)=\sqrt{{\left(x_{i}-x_{j}\right)}^{T}M\left(x_{i}-x_{j}\right)}$. When M is diagonal, this reduces to learning feature-specific weights, providing interpretable feature weights (17).

Despite its promise, metric learning has seen limited application in microbiome beta diversity analysis. Previous work has explored metric learning for clinical prediction tasks (19), but not specifically for statistical inference in community composition analysis where rigorous type I error control is essential.

### Study objectives

We developed Metric Learning for Statistical Inference (MeLSI) to bridge the gap between adaptive machine learning approaches and rigorous statistical inference for microbiome beta diversity and community composition analysis. Our specific objectives were to (i) design an ensemble metric learning framework that learns data-adaptive distance metrics for PERMANOVA and ordination while preventing overfitting, (ii) integrate metric learning with permutation testing to ensure valid statistical inference, (iii) comprehensively validate type I error control, statistical power, scalability, parameter sensitivity, and computational efficiency, (iv) demonstrate practical utility on real microbiome data sets, and (v) provide interpretable learned metric weights to identify biologically relevant taxa driving community separation.

This paper presents the MeLSI framework, comprehensive validation results, and discussion of its implications for microbiome beta diversity research.

## MATERIALS AND METHODS

### Overview of the MeLSI framework

MeLSI integrates metric learning with permutation-based statistical inference through two main phases.

#### Phase 1: metric learning

1. Apply conservative pre-filtering to focus on high-variance features.
2. For each of B weak learners:
  - Bootstrap sample the data.
  - Subsample features.
  - Optimize metric matrix *M* via gradient descent.
3. Combine weak learners via performance-weighted ensemble averaging.
4. Compute robust distance matrix using eigenvalue decomposition.

#### Phase 2: statistical inference

1. Calculate observed *F*-statistic using the learned metric.
2. Generate null distribution via permutation testing (relearn metric on each permutation).
3. Compute permutation-based *P*-value.

Each component addresses specific challenges in microbiome data analysis while maintaining statistical validity. The following sections formalize the mathematical framework and detail each algorithmic component, organized by phase.

### Phase 1: metric learning

#### Problem formulation

Let $X\in R^{n\times p}$ denote a feature abundance matrix with *n* samples and *p* taxa (features), and let $y=\left(y_{1},\ldots ,y_{n}\right)$ denote group labels. Our goal is to learn a distance metric optimized for separating groups defined by y while ensuring valid statistical inference.

We parameterize the distance metric using a diagonal positive semi-definite matrix $M\in R^{p\times p}$, where *M_jj_* represents the learned metric weight of feature *j*. The learned Mahalanobis distance between samples *i* and *k* is:


$$d_{M}\left(x_{i},x_{k}\right)=\sqrt{{\left(x_{i}-x_{k}\right)}^{T}M\left(x_{i}-x_{k}\right)}$$

For diagonal M, this simplifies to a weighted Euclidean distance:


$$d_{M}\left(x_{i},x_{k}\right)=\sqrt{\sum_{j}M_{jj}{\left(x_{ij}-x_{kj}\right)}^{2}}$$

#### Conservative pre-filtering

To improve computational efficiency and reduce noise, MeLSI applies conservative variance-based pre-filtering. For pairwise comparisons, we calculate a pre-filtering score combining mean differences and variance:


$$I_{j}=\frac{\left|\mu_{1j}-\mu_{2j}\right|}{\sqrt{\sigma_{1j}^{2}+\sigma_{2j}^{2}}}$$

where $\mu_{1j}$ and $\mu_{2j}$ are the mean abundances of feature *j* in groups 1 and 2, and $\sigma_{1j}^{2}$ and $\sigma_{2j}^{2}$ are their variances. We retain the top 70% of features by this pre-filtering score, maintaining high statistical power while reducing dimensionality. This pre-filtering score $I_{j}$ is used solely for feature selection and is distinct from the learned metric weights *M_jj_*, which are optimized during metric learning (see section Optimization objective) and represent each feature’s contribution to the final distance metric.

For multi-group comparisons (three or more groups), we use ANOVA *F*-statistics to rank features and apply the same 70% retention threshold. These statistics serve as ranking heuristics to order features by between-group differences relative to within-group variation; no distributional assumptions are required because all statistical inference uses permutation testing. Critically, this pre-filtering is applied consistently to both observed and permuted data during null distribution generation to avoid bias.

#### Ensemble learning with weak learners

MeLSI constructs an ensemble of *B* weak learners (default B=30) to improve robustness and prevent overfitting. For each weak learner *b*:

1. Bootstrap sampling: draw *n* samples with replacement from the original data to create a bootstrap data set $\left(X_{b},y_{b}\right)$.
2. Feature subsampling: randomly select $m=\lfloor p\times m_{frac}\rfloor$ features (default $m_{frac}=0.8$) without replacement.
3. Metric optimization: learn $M_{b}$ on the bootstrapped, subsampled data.

The combination of bootstrap sampling (sample-level randomness) and feature subsampling (feature-level randomness) ensures diversity among weak learners, reducing overfitting risk (20).

#### Optimization objective

For each weak learner, we optimize M to maximize between-group distances while minimizing within-group distances. For a two-group comparison (groups *G*_1_ and *G*_2_), we maximize the objective:


$$F\left(M\right)=\frac{1}{|G_{1}||G_{2}|}\sum_{i\in G_{1}}\sum_{k\in G_{2}}d_{M}{\left(x_{i},x_{k}\right)}^{2}-\frac{1}{2|G_{1}{|}^{2}}\sum_{i,j\in G_{1}}d_{M}{\left(x_{i},x_{j}\right)}^{2}-\frac{1}{2|G_{2}{|}^{2}}\sum_{i,j\in G_{2}}d_{M}{\left(x_{i},x_{j}\right)}^{2}$$

This objective encourages large between-group distances and small within-group distances, analogous to maximizing the *F*-ratio in ANOVA. This formulation is inspired by standard metric learning objectives that maximize between-class to within-class distance ratios (16, 17), adapted here for direct compatibility with PERMANOVA’s *F*-statistic framework.

#### Gradient-based optimization

Each weak learner optimizes its metric matrix M using stochastic gradient descent, sampling within-group and between-group pairs to compute gradients that maximize between-group distances while minimizing within-group distances. We use an adaptive learning rate $\eta_{t}=\eta_{0}/(1+0.1t)$ (default $\eta_{0}=0.1$) and constrain $M_{jj}\geq 0.01$ to ensure positive definiteness. Early stopping monitors F-statistics every 20 iterations, terminating if performance stagnates (no improvement for five consecutive checks) to prevent overfitting.

#### Ensemble averaging with performance weighting

After training all weak learners, we combine them into a final ensemble metric $M_{ensemble}$ using performance-weighted averaging:


$$M_{ensemble}=\sum_{b}w_{b}M_{b}$$

where weights are normalized *F*-statistics:


$$w_{b}=\frac{F_{b}}{\sum_{b^{`}}F_{b^{`}}}$$

and *F_b_* is the PERMANOVA *F*-statistic achieved by weak learner *b* on its bootstrap sample. This weighting scheme emphasizes better-performing learners while maintaining diversity.

#### Robust distance calculation

To ensure numerical stability, we compute the learned Mahalanobis distance using eigenvalue decomposition:

1. Compute eigendecomposition: $M_{ensemble}=V\Lambda V^{T}$, where V is the matrix of eigenvectors and Λ is the diagonal matrix of eigenvalues.
2. Enforce positive eigenvalues: $max(\Lambda_{ii},10^{-6})\to \Lambda_{ii}$.
3. Compute $M^{-1/2}=V\Lambda^{-1/2}V^{T}$.
4. Transform data: $Y=XM^{-1/2}$.
5. Calculate Euclidean distances in transformed space: $d_{M}=||y_{i}-y_{k}|{|}_{2}$.

This approach is more numerically stable than direct matrix inversion, particularly for high-dimensional data.

### Phase 2: statistical inference

Phase 2 focuses on statistical inference using the learned metric from phase 1. We compute *P*-values through permutation testing to ensure valid statistical inference.

### Statistical inference via permutation testing

#### Test statistic

We use the PERMANOVA *F*-statistic as our test statistic (5):


$$F_{obs}=\frac{SS_{between}/\left(k-1\right)}{SS_{within}/\left(n-k\right)}$$

where SS_between_ is the between-group sum of squares, SS_within_ is the within-group sum of squares, *k* is the number of groups, and *n* is the total number of samples. This statistic measures how well the learned metric separates groups relative to within-group variation.

#### Null distribution generation

To compute valid *P*-values, we generate a null distribution under the hypothesis of no group differences:

1. Permute group labels: random permutation of $y\to y_{perm}$.
2. Apply identical pre-filtering to permuted data.
3. Learn metric $M_{perm}$ on $\left(X_{filtered},y_{perm}\right)$ using the full MeLSI algorithm (repeating phase 1: pre-filtering, ensemble construction, and metric optimization).
4. Calculate $F_{perm}$ on $\left(X_{filtered},y_{perm}\right)$ with $M_{perm}$.
5. Repeat steps 1–4 for $n_{perms}$ permutations (default $n_{perms}=200$).

This approach ensures that the null distribution accurately reflects the variability introduced by the metric learning procedure itself, avoiding anticonservative (inflated type I error) inference.

#### *P*-value calculation

The permutation-based *P*-value is computed as follows:


$$P=\frac{\sum I\left(F_{perm}\geq F_{obs}\right)+1}{n_{perms}+1}$$

where I is the indicator function. The “+1” terms provide a small-sample correction ensuring $P\geq 1/\left(n_{perms}+1\right)$ (21).

### Validation experiments

We conducted comprehensive validation experiments to assess the following:

1. Type I error control and statistical power: performance on null data (no true group differences) and ability to detect true effects of varying magnitude across synthetic data sets (see “Type I error control”).
2. Scalability: performance across varying sample sizes and dimensionalities (see “Scalability analysis”).
3. Parameter sensitivity: robustness to hyperparameter choices (see “Parameter sensitivity analysis”).
4. Feature correlation robustness: performance under varying levels of feature correlation (see “Feature correlation robustness”).
5. Pre-filtering value: benefit of conservative feature pre-filtering (see “Pre-filtering”).
6. Real data validation: comparative performance against standard distance metrics on Atlas1006, DietSwap, and SKIOME data sets (see “Real data validation”).
7. Biological interpretability: learned metric weights and visualization (see “Learned metric weights”).
8. Computational performance: runtime characteristics on standard hardware (see “Computational performance”).

#### Synthetic data generation

Synthetic data sets were generated using negative binomial count distributions to mimic microbiome abundance profiles. For each experiment, we drew counts as *X_ij_* ∼ NB (µ = 30, size = 0.8) and set values smaller than three to zero to induce sparsity. Unless otherwise noted, we simulated *n* = 100 samples and *p* = 200 taxa split evenly across two groups. To introduce signal, we multiplied a subset of taxa in the first group by fold changes of 1.5 (5 taxa, “small” effect), 2.0 (10 taxa, “medium” effect), or 3.0 (20 taxa, “large” effect). Sample size (*n*) and dimensionality (*p*) were varied in the scalability experiments (see “Scalability analysis”), while null data sets were formed by random label permutations or by shuffling labels in real data without adding signal.

#### Real data sources

Real microbiome data sets included the following:

1. Atlas1006 (22): 1,114 Western European adults with 123 genus-level taxa from HITChip microarray technology. Analysis compared males (*n* = 560) versus females (*n* = 554).
2. DietSwap (23): 74 stool samples from African American adults participating in a short-term dietary intervention. We analyzed the time point-within-group baseline samples (timepoint.within.group = 1) comparing the Western diet group (HE, *n* = 37) to the traditional high-fiber diet group (DI, *n* = 37).
3. SKIOME (PRJNA554499): 511 skin microbiome samples across three groups (Atopic Dermatitis, Healthy, Psoriasis) with 1,856 taxa, used for multi-group validation across a different body site.

Data were preprocessed using centered log ratio (CLR) transformation for Euclidean distance analyses to address compositionality (11, 24). CLR transformation converts relative abundances to log ratios, making the data suitable for Euclidean distance while preserving relative relationships between taxa. CLR treats abundance ratios more equitably than count-based metrics, which can be dominated by highly abundant taxa. However, CLR transformation may attenuate large fold change signals compared to count-based metrics (Bray-Curtis, UniFrac), as evidenced by our results showing higher empirical power for count-based methods at medium and large effects (Table 2). CLR is particularly appropriate when signals are distributed across multiple taxa rather than concentrated in highly abundant taxa, and when interpretability through learned metric weights is prioritized. Bray-Curtis dissimilarity, Jaccard, and UniFrac distances were computed on raw count data, as these metrics are inherently designed to handle compositional data (7, 25). Prevalence filtering (retaining features present in ≥10% of samples) is an optional preprocessing step distinct from MeLSI’s variance-based pre-filtering; when applied, prevalence filtering removes rare taxa before analysis, while MeLSI’s pre-filtering focuses on variance-based feature selection after preprocessing.

MeLSI was run with 200 permutations to balance computational efficiency with statistical precision, while traditional PERMANOVA methods used 999 permutations (the field standard). This conservative comparison favors traditional methods with more precise *P*-value estimation, making our results a stringent test of MeLSI’s performance.

#### Comparison methods

MeLSI was compared against standard PERMANOVA analyses using five fixed distance metrics:

1. Euclidean distance: standard Euclidean distance calculated on CLR-transformed data, treating all features equally.
2. Bray-Curtis dissimilarity: count-based dissimilarity metric that accounts for relative abundances.
3. Jaccard dissimilarity: binary (presence/absence) dissimilarity metric.
4. Weighted UniFrac: phylogenetically informed distance metric using abundance-weighted branch lengths (requires phylogenetic tree).
5. Unweighted UniFrac: phylogenetically informed distance metric using presence/absence of taxa along phylogenetic branches (requires phylogenetic tree).

To ensure a robust comparison, weighted/unweighted UniFrac were provided with random phylogenetic trees for synthetic benchmarks. UniFrac was not evaluated on real-world data sets because the publicly available data set objects for Atlas1006, DietSwap, and SKIOME lack phylogenetic trees.

### Multi-group extensions

For studies with three or more groups, MeLSI provides an omnibus test that jointly evaluates differences across all groups, with *post hoc* pairwise comparisons when significant. *P*-values are adjusted for multiple testing using the Benjamini-Hochberg false discovery rate (FDR) procedure (26). The statistical framework (permutation testing, type I error control) is identical to two-group analyses, ensuring valid inference regardless of group number. Real-world validation on the SKIOME skin microbiome data set (three groups, 511 samples) demonstrates utility beyond two-group comparisons (see Results).

### Implementation and computational considerations

MeLSI is implemented in R (version ≥ 4.0) as an open-source package. Key dependencies include vegan (27) for PERMANOVA calculations, ggplot2 (28) for visualization, and base R for matrix operations. The algorithm is parallelizable across permutations and weak learners, though the current implementation is serial.

Time complexity is *O*(*n*²*p*²*B*·*n*_perms) in the worst case, but conservative pre-filtering reduces effective dimensionality, and early stopping in gradient descent reduces iteration counts. For typical microbiome data sets (*n* < 500, *p* < 1,000), analysis completes in minutes on standard hardware.

## RESULTS

Our validation strategy follows a rigorous progression from statistical validity to biological utility. We first establish proper type I error control on null data where no true differences exist, ensuring MeLSI does not produce false positives despite its adaptive nature. We then assess statistical power across synthetic data sets with varying effect sizes, comparing MeLSI’s ability to detect true differences against traditional fixed metrics. Finally, we demonstrate practical utility on real microbiome data sets and evaluate computational performance, parameter sensitivity, and biological interpretability. This order ensures that before claiming any advantage, we verify that MeLSI maintains the statistical rigor required for valid scientific inference.

### Type I error control

Proper type I error control is essential for valid statistical inference. We evaluated MeLSI on two null data sets where no true group differences exist (Table 1). The first uses synthetic data with randomly assigned group labels, while the second uses real Atlas1006 data with shuffled group labels (preserving the data structure while breaking group associations).

**TABLE 1:** Type I error control on null data^*a*^

Data set type	*n*	MeLSI type I	Euclidean type I	Bray-Curtis type I	Jaccard type I	Weighted UniFrac type I	Unweighted UniFrac type I
Null synthetic	50	5%	7%	7%	6%	3%	4%
Null synthetic	100	4%	3%	2%	5%	2%	4%
Null synthetic	200	3%	0%	5%	2%	2%	4%
Null real shuffled	50	3%	4%	4%	6%	6%	9%
Null real shuffled	100	4%	4%	4%	3%	4%	4%
Null real shuffled	200	6%	4%	4%	2%	4%	1%

^*a*^ *n*, sample size; type I, empirical type I error rate (percentage of simulations with *P* < 0.05). Results based on 100 simulations per condition.

Across all conditions, MeLSI maintained proper type I error control, with empirical rejection rates near the nominal 5% level (range: 3%–6%). All traditional methods also maintained appropriate error rates (range: 0%–9%). The permutation testing framework properly accounts for the flexibility of learned metrics, ensuring that MeLSI’s adaptive approach does not inflate false positive rates.

### Performance across synthetic and real data sets

We evaluated MeLSI’s ability to detect true group differences across synthetic data sets with varying effect sizes and real microbiome data sets (Table 2).

**TABLE 2:** Empirical power (%) across methods, effect sizes, and sample sizes^*a*^

Effect size	*n*	MeLSI	Euclidean	Bray-Curtis	Jaccard	UniFrac	UW. UniFrac
Small	50	6	8	20	0	10	6
Small	100	10	8	20	6	8	4
Small	200	16	16	54	6	20	4
Medium	50	16	32	74	0	32	2
Medium	100	50	52	100	4	82	4
Medium	200	96	100	100	0	100	2
Large	50	84	98	100	10	98	8
Large	100	100	100	100	4	100	4
Large	200	100	100	100	6	100	6

^*a*^ *n*, sample size per group; power, percentage of 50 simulations with *P* < 0.05. MeLSI and Euclidean use CLR-transformed data; Bray-Curtis, Jaccard, and UniFrac use raw counts. MeLSI used 200 internal permutations; traditional methods used 999 permutations. See Tables S1 and S2 for signal taxon recovery metrics and per-method *F*-statistics.

MeLSI demonstrated competitive detection across all conditions. The most informative comparison is MeLSI versus Euclidean distance, as both operate on CLR-transformed data. MeLSI matched Euclidean power at small effects and showed comparable performance through large effects, where both reached 100% power at *n* ≥ 100. This equivalence confirms that MeLSI’s adaptive weighting does not come at a cost to detection relative to its natural baseline. At large effects with sufficient sample size (*n* ≥ 100), all abundance-sensitive methods (MeLSI, Euclidean, Bray-Curtis, weighted UniFrac) converged to 100% power, reflecting the expected behavior that sufficiently strong signals are detectable by any reasonable method. Bray-Curtis and weighted UniFrac showed higher power at medium effects, attributable to their direct operation on raw count data rather than CLR-transformed data; this advantage does not reflect a difference in metric quality but rather a difference in input scale. Jaccard and Unweighted UniFrac showed near-zero power because these presence/absence metrics cannot detect fold change effects: multiplying the abundance of a taxon already present in both groups does not change its binary profile. PERMANOVA *F*-statistics from different distance metrics are not directly comparable because each metric defines a distinct geometric space, so cross-metric comparisons should be interpreted with caution. Beyond detection, MeLSI provides learned feature weights that identify which taxa drive group separation (Table S1), an output that no fixed distance metric can supply.

#### Signal taxa recovery

MeLSI’s learned feature weights identify which taxa drive group differences, providing interpretability that fixed metrics cannot offer. Table S1 reports recovery metrics across all simulated conditions. At small effects (1.5× fold change in five taxa), recovery was modest, with precision at 5 ranging from 0.104 to 0.148 and AUC-ROC from 0.641 to 0.673 across sample sizes. At medium effects (2.0× fold change in 10 taxa), performance improved substantially with sample size, with precision at 5 increasing from 0.356 (*n* = 50) to 0.660 (*n* = 200) and AUC-ROC from 0.733 to 0.842. At large effects (3.0× fold change in 20 taxa), recovery was strong: precision at 5 reached 0.876–1.000, mean rank decreased to 14.4–26.2 out of 200 taxa, and AUC-ROC reached 0.858–0.960. For context, random assignment would yield precision at 5 of 0.025–0.100 and AUC-ROC of 0.500, so even modest small-effect recovery represents meaningful signal detection above chance. MeLSI’s learned weights are most reliable as a feature ranking tool when effects are moderate to large, which are precisely the conditions where identification of specific taxa driving group separation is most biologically actionable.

#### Scalability analysis

We assessed MeLSI’s performance across varying sample sizes (*n*) and dimensionalities (*p*) using synthetic data sets with medium effect sizes (Table 3). For sample size scaling, we fixed *p* = 200 taxa and varied *n* from 20 to 500. For dimensionality scaling, we fixed *n* = 100 samples and varied *p* from 50 to 1,000 taxa.

**TABLE 3:** Scalability across sample size and dimensionality^*a*^

Parameter	*n*	*p*	MeLSI *F*	MeLSI time
Varying *n* (*p* = 200)				
*n* = 20	20	200	1.132	486.9
*n* = 50	50	200	1.277	457.9
*n* = 100	100	200	1.497	513.3
*n* = 200	200	200	1.836	739.5
*n* = 500	500	200	2.511	2,055.8
Varying *p* (*n* = 100)				
*p* = 50	100	50	1.666	244.8
*p* = 100	100	100	1.670	337.5
*p* = 200	100	200	1.470	523.4
*p* = 500	100	500	1.375	1,829.0
*p* = 1,000	100	1,000	1.331	8,633.0

^*a*^ *n*, sample size; *p*, number of taxa/features; MeLSI *F*, MeLSI PERMANOVA *F*-statistic; time, computation time in seconds. Values shown as mean across 10 simulations per condition. See Table S3 for individual method *F*-statistics.

MeLSI’s *F*-statistics increased monotonically with sample size, demonstrating appropriate signal detection with larger data sets. Computation time scaled as *O*(*n*²) with sample size and *O*(*p*²) with dimensionality, but pre-filtering substantially mitigates the dimensionality scaling. For very high-dimensional data sets (*p* > 1,000), we recommend pre-filtering, feature aggregation, or traditional methods if interpretability is not prioritized.

#### Parameter sensitivity analysis

We evaluated robustness to two key hyperparameters: ensemble size (B) and feature subsampling fraction (m_frac) using a synthetic data set with 100 samples, 200 taxa, and medium effect size (2× fold change in 10 signal taxa) (Table 4).

**TABLE 4:** Parameter sensitivity analysis^*a*^

Parameter	Value	*F*-statistic	*P*-value	Time (s)
Ensemble size (*B*)	1	1.365	0.421	32.9
	10	1.543	0.094	233
	20	1.538	0.089	419.8
	30	1.530	0.091	576.8
	50	1.529	0.093	760
	100	1.528	0.102	1,284.1
Feature fraction (m_frac)	0.5	1.578	0.093	405.2
	0.7	1.551	0.083	523.7
	0.8	1.530	0.091	578.2
	0.9	1.517	0.097	630.3
	1.0	1.498	0.100	666.7

^*a*^ *B*, ensemble size (number of weak learners); m_frac, feature subsampling fraction; *F*, PERMANOVA *F*-statistic; time, computation time in seconds. Values shown as mean across 25 replications per parameter value. See Table S4 for standard deviations.

*F*-statistics remained stable across ensemble sizes (*B* = 10–100), with the single-learner baseline (*B* = 1) showing substantially higher variance, demonstrating that ensemble learning reduces variance and prevents overfitting. Performance varied modestly across feature fractions (m_frac = 0.5–1.0). The default settings (*B* = 30, m_frac = 0.8) provide a good balance between performance and computational cost.

### Feature correlation robustness

A critical concern for microbiome data analysis is that taxa are not independent but exhibit correlations due to ecological relationships (e.g., co-occurring taxa in microbial communities). To validate MeLSI’s robustness to feature correlation, we evaluated performance across four correlation levels: none (*r* = 0), low (*r* = 0.3), moderate (*r* = 0.6), and high (*r* = 0.8), using 50 simulations per condition (200 total simulations) with synthetic data sets containing 100 samples, 200 taxa, and medium effect size (2× fold change in 10 signal taxa) (Table 5). Correlation was introduced using a block structure: the 200 taxa were divided into 10 blocks of 20 taxa each, with uniform pairwise correlation imposed among taxa within each block and independence between blocks. This design mimics realistic microbiome correlation patterns, where functionally related or co-occurring taxa (e.g., within the same ecological guild or metabolic pathway) exhibit positive correlation, while taxonomically distant groups remain independent. Within each block, correlated multivariate normal noise was generated (using Cholesky decomposition) and added to log-transformed abundances, scaled to preserve the original signal structure. Signal taxa were randomly distributed across blocks, so some blocks contained signal taxa and others did not, reflecting the realistic scenario where correlated taxa may or may not include differentially abundant species.

**TABLE 5:** Effect of feature correlation on MeLSI performance^*a*^

Correlation level	Correlation value	*n*	MeLSI power (%)	MeLSI *F*	Precision at 10	Recall at 10	AUC-ROC
None	0.0	50	50	1.512	0.392	0.392	0.817
Low	0.3	50	42	1.481	0.348	0.348	0.788
Moderate	0.6	50	46	1.498	0.356	0.356	0.783
High	0.8	50	44	1.507	0.368	0.368	0.769

^*a*^ *n*, number of simulations per correlation level; *F*, PERMANOVA *F*-statistic (mean across 50 simulations); Precision at 10, proportion of top 10 features that are true signals; Recall at 10, proportion of true signals found in top 10 features; AUC-ROC, area under receiver operating characteristic curve. Sample size fixed at 100 samples, 200 taxa, medium effect size. See Table S5 for individual method comparisons.

MeLSI demonstrated robust performance across correlation levels, maintaining stable *F*-statistics (±1.7% variation: *F* = 1.512 at *r* = 0, *F* = 1.481 at *r* = 0.3, *F* = 1.498 at *r* = 0.6, *F* = 1.507 at *r* = 0.8) and consistent statistical power (50%, 42%, 46%, and 44%, respectively). The stability of *F*-statistics demonstrates that MeLSI effectively handles correlated features without performance degradation. Feature recovery metrics also remained stable: precision at 10 (0.392, 0.348, 0.356, 0.368) and AUC-ROC (0.817, 0.788, 0.783, 0.769) showed minimal variation across correlation levels, confirming that MeLSI’s ability to identify true signal taxa is maintained even when taxa exhibit high correlation. These results indicate that the ensemble approach, which aggregates signal across multiple learners with different bootstrap samples and feature subsets, naturally accommodates correlated features without requiring explicit correlation modeling.

### Pre-filtering analysis

We evaluated the benefit of conservative pre-filtering by comparing MeLSI with and without this step using synthetic data sets (*n* = 100 samples per condition) with varying effect sizes and dimensionalities (small: 1.5× fold change in five taxa, *p* = 500; medium: 2.0× in 10 taxa, *p* = 200; large: 3.0× in 20 taxa, *p* = 100) and high sparsity (70% zero-inflated features) (Table 6).

**TABLE 6:** Benefit of conservative pre-filtering^*a*^

Effect	Features	Filter *F*	Filter power	No filter *F*	No filter power	Delta *F*	Delta time
Small	500	1.756	100%	1.281	4%	+37.1%	+39.8%
Medium	200	1.831	94%	1.337	14%	+36.9%	+18.0%
Large	100	1.928	84%	1.416	14%	+36.1%	+16.5%

^*a*^ Effect, effect size category (small: 1.5× fold change in five taxa; medium: 2.0× in 10 taxa; large: 3.0× in 20 taxa); features, number of taxa; *F*, PERMANOVA *F*-statistic (mean across 50 simulations); power, empirical statistical power (percentage of simulations with *P* < 0.05); Filter, with pre-filtering (top 70% by importance score); no filter, without pre-filtering; delta *F*, percent change in *F*-statistic; delta time, percent change in computation time (positive = time savings). Results based on 50 simulations per condition.

Variance-based pre-filtering (retaining the top 70% of features by importance score) demonstrated substantial benefits across all effect sizes. Pre-filtering improved *F*-statistics by 36%–37% across all effect sizes, increasing power from 4%–14% to 84-100% for small effects. Time savings ranged from 16.5% to 39.8%, increasing with dimensionality. The variance-based importance score ($I_{j}=|\mu_{1j}-\mu_{2j}|/\sqrt{\sigma_{1j}^{2}+\sigma_{2j}^{2}}$) efficiently identifies features with large between-group differences relative to within-group variation. Pre-filtering is particularly valuable when signal is concentrated in a subset of features, focusing metric learning on the most informative taxa while reducing computational burden.

### Real data validation

To evaluate MeLSI’s utility in real-world applications, we analyzed three published microbiome data sets: Atlas1006 (sex-associated differences), DietSwap (dietary intervention), and SKIOME (multi-group skin microbiome validation).

#### Atlas1006 data set

On the Atlas1006 data set (1,114 Western European adults, male vs female), all methods except Jaccard detected significant sex-associated community differences: MeLSI (*F* = 4.841, *P* = 0.005), Euclidean (*F* = 4.711, *P* = 0.001), Bray-Curtis (*F* = 4.442, *P* = 0.001), Jaccard (*F* = 1.791, *P* = 0.144). These results are consistent with previously documented microbiome variation between males and females (29, 30). *F*-statistics from different distance metrics are not directly comparable because each defines a distinct geometric space; significance (*P*-values) provides the appropriate basis for comparison.

##### Learned metric weights

**Fig 1:**
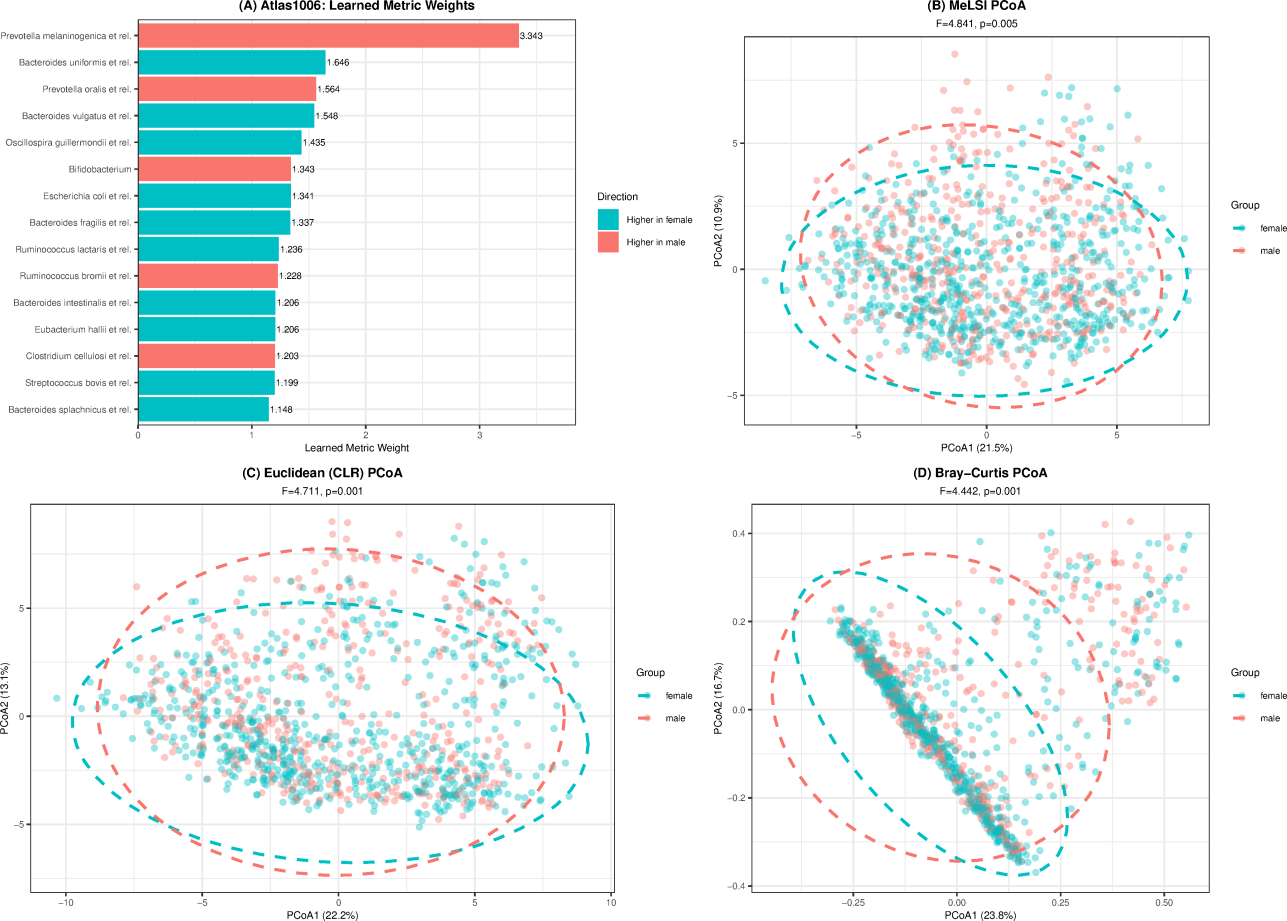
Atlas1006 data set (male vs female, *n* = 1,114). (**A**) Top 15 taxa ranked by MeLSI learned metric weights, colored by directionality (group with higher mean CLR abundance). Taxa from Bacteroidaceae, Lachnospiraceae, and Ruminococcaceae families show the strongest contributions. (**B**) PCoA ordination using MeLSI learned distance (*F* = 4.841, *P* = 0.005). (**C**) PCoA ordination using Euclidean distance on CLR-transformed data (*F* = 4.711, *P* = 0.001). (**D**) PCoA ordination using Bray-Curtis dissimilarity (*F* = 4.442, *P* = 0.001). Dashed ellipses show 95% confidence ellipses. Note: PCoA1/PCoA2 axes are on different scales across panels because each distance metric defines a distinct geometric space.

MeLSI provides interpretable learned metric weights. For the Atlas1006 data set, the learned metric assigned highest weights to genera in the families Bacteroidaceae, Lachnospiraceae, and Ruminococcaceae, taxonomic groups previously associated with sex differences in gut microbiome composition (30, 31). Figure 1 displays the top 15 taxa by learned feature weight.

**Fig 2:**
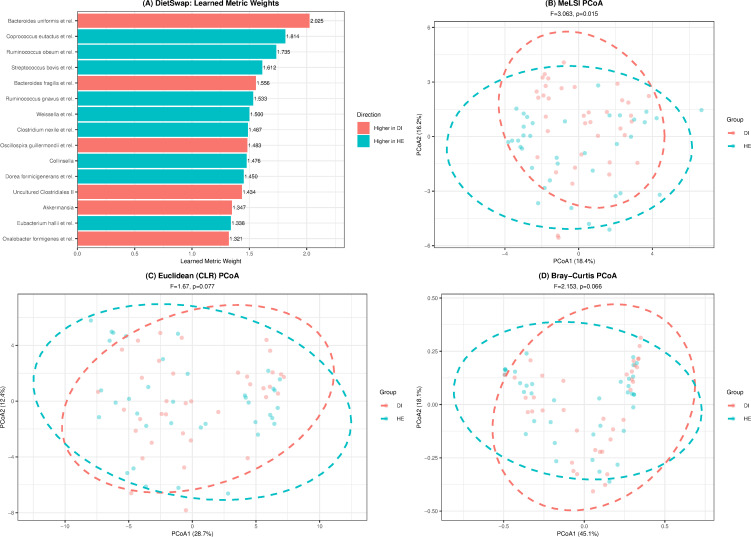
DietSwap data set (Western vs high-fiber diet, *n* = 74). (**A**) Top 15 taxa by MeLSI learned metric weights, colored by directionality. (**B**) PCoA ordination using MeLSI learned distance (*F* = 3.063, *P* = 0.015). (**C**) PCoA ordination using Euclidean distance on CLR-transformed data (*F* = 1.670, *P* = 0.077). (**D**) PCoA ordination using Bray-Curtis dissimilarity (*F* = 2.153, *P* = 0.066). Dashed ellipses show 95% confidence ellipses. MeLSI was the only method achieving significance at α = 0.05.

**Fig 3:**
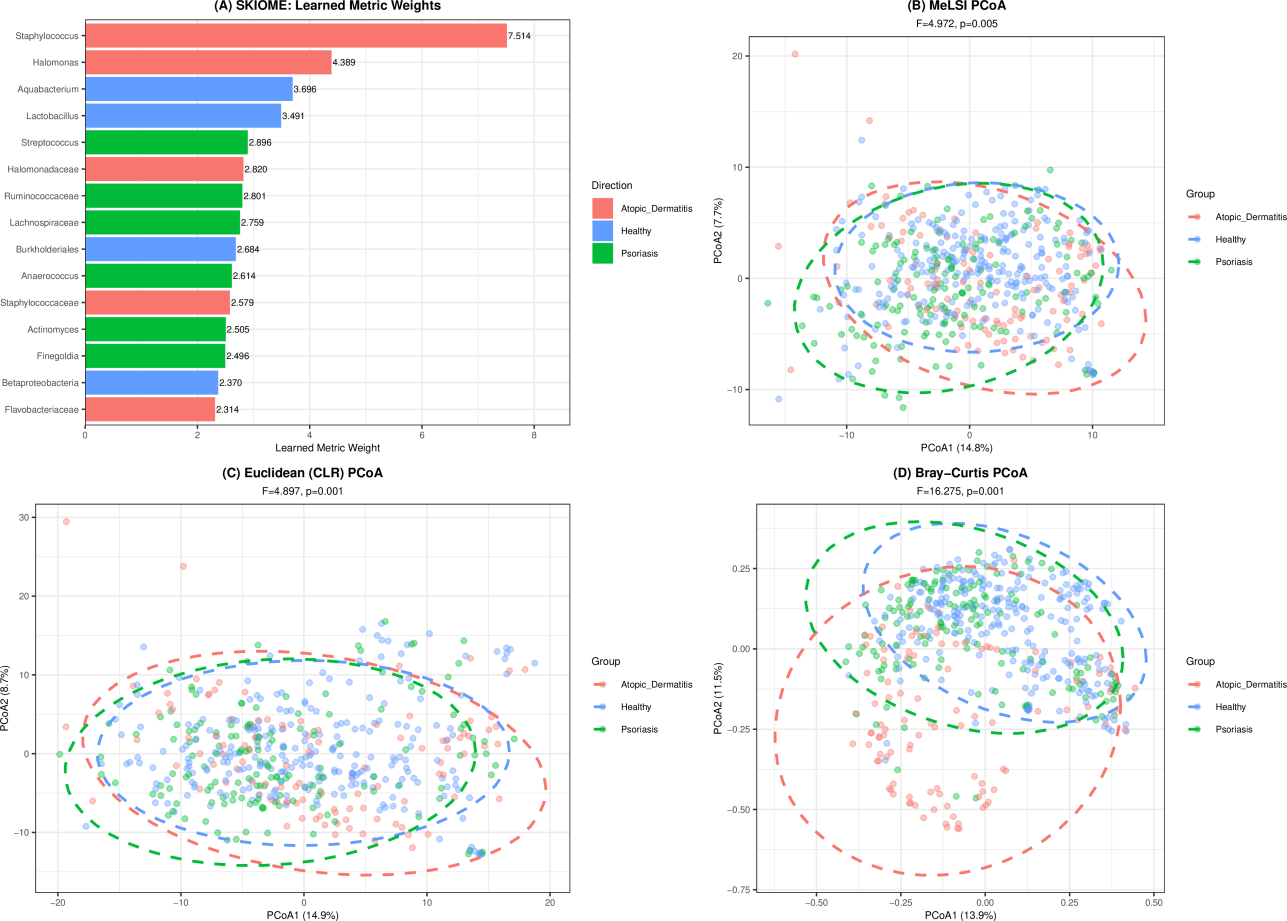
SKIOME multi-group validation (atopic dermatitis, healthy, psoriasis; *n* = 511). (**A**) Top 15 taxa by MeLSI learned metric weights, colored by group with highest mean abundance. (**B**) PCoA ordination using MeLSI learned distance (*F* = 4.972, *P* = 0.005). (**C**) PCoA ordination using Euclidean distance on CLR-transformed data (*F* = 4.897, *P* = 0.001). (**D**) PCoA ordination using Bray-Curtis dissimilarity (*F* = 16.275, *P* = 0.001). Dashed ellipses show 95% confidence ellipses. All methods detected significant differences; Bray-Curtis showed the strongest visual separation.

The diagonal elements of the learned metric matrix M represent the learned metric weights: higher values indicate taxa that contribute more to group separation. These metric weights are distinct from the pre-filtering scores $I_{j}$ used for feature selection. MeLSI automatically calculates directionality and effect sizes on CLR-transformed data. Directionality is determined by identifying which group has the higher mean abundance on CLR-transformed data. Effect size is reported as the log_2_ fold change computed from CLR-transformed group means: ${log}_{2}(\mu_{\text{CLR,1}}/\mu_{\text{CLR,2}})$. The learned distance matrices can also be used for PCoA ordination to visualize group separation (Fig. 1 to 3), enabling direct visual comparison with traditional metrics on the same data sets.

### DietSwap data set

On the DietSwap data set (Western vs high-fiber diets), MeLSI was the only method to detect a significant community difference at α = 0.05: MeLSI (*F* = 3.063, *P* = 0.015), Bray-Curtis (*F* = 2.153, *P* = 0.066), Jaccard (*F* = 1.921, *P* = 0.100), Euclidean (*F* = 1.670, *P* = 0.077). Phylogenetic methods (weighted/unweighted UniFrac) were not evaluated because the publicly available data set object lacks a phylogenetic tree. This demonstrates a case where MeLSI’s adaptive weighting captures diet-induced compositional shifts that fixed metrics detect only marginally.

#### Learned metric weights

For the DietSwap data set, MeLSI’s learned metric weights identified taxa including Akkermansia and Oxalobacter as key drivers of diet-induced community differences. Figure 2 displays the top 15 taxa by learned feature weight alongside the PCoA ordination.

### SKIOME data set

To validate multi-group capability, we analyzed the SKIOME skin microbiome data set (PRJNA554499, 511 samples, three groups: Atopic_Dermatitis, Healthy, Psoriasis). MeLSI’s omnibus test detected significant differences (*F* = 4.972, *P* = 0.005), as did Euclidean distance (*F* = 4.897, *P* = 0.001), Bray-Curtis (*F* = 16.275, *P* = 0.001), and Jaccard (*F* = 11.058, *P* = 0.001). All pairwise comparisons remained significant after FDR correction (*P* = 0.005 for all pairs). The most informative comparison is MeLSI versus Euclidean, as both operate in CLR-transformed space: MeLSI (*F* = 4.972) performed essentially identically to unweighted Euclidean (*F* = 4.897). This equivalence is itself a biologically meaningful finding: it indicates that the signal in the SKIOME data set is broadly distributed across many taxa rather than concentrated in a small subset. In such cases, a weighted metric offers no detection advantage, and MeLSI correctly learns this by assigning relatively uniform weights rather than artificially inflating a few taxa. This reflects statistical integrity, not a limitation. Bray-Curtis and Jaccard yield substantially larger pseudo-*F* statistics because count-based and presence/absence metrics define fundamentally different geometric spaces; these values are not directly comparable to CLR-based *F*-statistics. Regardless, while Bray-Curtis provides only an omnibus *P*-value, MeLSI additionally provides learned feature weights identifying which taxa drive group separation (Fig. 3A), enabling biological interpretation within a single unified framework.

#### Learned metric weights

Figure 3 displays learned metric weights and PCoA ordination, demonstrating MeLSI’s interpretability for multi-group analyses. The learned metric weights identified *Staphylococcus* (family Staphylococcaceae) as the most influential taxon, consistent with its known role in atopic dermatitis pathogenesis. This validates MeLSI’s utility beyond two-group comparisons and across different body sites (skin vs gut microbiome).

### Computational performance

Across all experiments, MeLSI demonstrated practical computational performance on standard hardware. Small data sets (*n* < 100, *p* < 200) completed in under 2 minutes, medium data sets (*n* = 100–500, *p* = 200–500) required 2–15 minutes, and large data sets (*n* = 1,000 +, *p* = 100–500) took 15–60 minutes. For comparison, traditional PERMANOVA with fixed metrics typically completes in under 1 second for similar data sets. MeLSI’s additional computation time is justified by its interpretability advantage: learned feature weights identify which taxa drive group separation, information that fixed distance metrics cannot provide. Pre-filtering improves *F*-statistics by 36%–37% while reducing computation time by 16%–40% (Table 6). For typical microbiome studies (*n* = 50–200, *p* = 100–500), MeLSI completes in 2–30 minutes (Table 3), representing a modest time investment relative to overall study timelines. For very large studies (*n* > 500) or when only rapid screening is needed without feature-level interpretation, traditional methods may be preferable.

## DISCUSSION

### Summary

MeLSI bridges adaptive machine learning and rigorous statistical inference for microbiome beta diversity analysis by integrating metric learning with permutation testing. Comprehensive validation demonstrates proper type I error control across 100 simulations per condition, with empirical rejection rates near the nominal 5% level (3%–6% across all conditions and sample sizes). On real data, MeLSI achieved significant detection on the DietSwap data set where all traditional metrics remained marginal (MeLSI *P* = 0.015 vs traditional *P* ≥ 0.066), and comparable significance on Atlas1006 and SKIOME. In synthetic benchmarks, Bray-Curtis and Weighted UniFrac showed higher empirical power than MeLSI at medium effect sizes (Table 2), reflecting the inherent advantage of count-based metrics for detecting fold-change signals in raw abundance data. This power difference is partly attributable to MeLSI using 200 permutations versus 999 for traditional methods; despite this conservative setting, MeLSI demonstrated comparable power to Euclidean distance (both CLR-based) while providing interpretability that no fixed metric offers.

MeLSI’s key innovation is interpretability: learned metric weights identify biologically relevant taxa (e.g., Bacteroidaceae, Lachnospiraceae, Ruminococcaceae in sex-associated differences), turning omnibus PERMANOVA results into actionable biological insights. The mechanism by which learned weights improve analysis is by assigning higher weights to taxa with strong between-group differences and lower weights to noise taxa; the weighted distance metric effectively increases the signal-to-noise ratio in the distance calculation. This concentrates the PERMANOVA analysis on the most informative features, analogous to how a domain expert might focus on key taxa, but learned directly from data rather than requiring prior knowledge. This weighting is particularly beneficial when a small subset of taxa drives group differences while many uninformative taxa add noise, as is typical in microbiome studies. MeLSI is recommended when (i) interpretability through feature weights is needed to identify biologically relevant taxa, (ii) traditional methods yield marginal results (*P*-values near 0.05), (iii) signals are distributed across multiple taxa rather than concentrated in highly abundant taxa, and (iv) the researcher seeks both a significance test and a ranked list of taxa driving group separation. Traditional methods (Bray-Curtis, UniFrac) are preferable for (i) large-scale screening studies where speed is critical, (ii) when only omnibus significance testing is needed without feature-level interpretation, and (iii) when fold -change signals are large and concentrated in abundant taxa. Critically, unlike prediction-focused machine learning (e.g., random forest, neural networks), MeLSI is an inference-focused approach: every learned metric undergoes rigorous permutation testing to ensure that *P*-values remain valid despite the adaptive nature of the method.

### Limitations and future work

MeLSI requires more computation time than fixed metrics (minutes vs seconds), reflecting the cost of learning optimal metrics through ensemble training and permutation testing. However, MeLSI’s computational time (2–30 minutes for typical data sets) is justified by substantial interpretability gains through learned feature weights, combined with a favorable power-time trade-off through pre-filtering (Table 6). For large-scale screening studies with thousands of samples, traditional methods may be more appropriate.

Synthetic validation focused on two-group comparisons, which represent the primary use case; multi-group synthetic validation would require duplicating all validation tables and is addressed through real-world multi-group validation on the SKIOME skin microbiome data set (three groups, 511 samples). The statistical framework (permutation testing, type I error control) is identical for two-group and multi-group analyses, ensuring valid inference regardless of group number.

The most immediate extensions are (i) regression and covariate adjustment to handle continuous outcomes and confounders (age, BMI, medication use), enabling integration with epidemiological frameworks, (ii) paired and longitudinal design support through blocked permutations (restricting permutations within subjects or pairs via the strata argument in PERMANOVA), and (iii) improved compositionality handling by learning metrics directly in compositional space using Aitchison geometry (24), potentially offering advantages for zero-inflated microbiome data.

MeLSI’s learned distance metrics are compatible with other distance-based ordination and hypothesis testing methods. The learned distances can be used with non-metric multidimensional scaling (32) and analysis of similarities (33), both of which operate on distance matrices and would benefit from MeLSI’s data-adaptive metrics. However, principal component analysis (PCA) is not compatible with MeLSI’s learned distances, as PCA relies on Euclidean distances computed in the original feature space and cannot accommodate the learned Mahalanobis distance structure.

### Software availability

MeLSI is freely available as a Bioconductor R package (https://bioconductor.org/packages/MeLSI/, installable via BiocManager::install(“MeLSI”)) at and at https://github.com/NathanBresette/MeLSI under the MIT license. The analysis reported here used MeLSI version 1.0.0; subsequent releases incorporate optimized code that reduce computation time with statistically identical results. The package includes comprehensive documentation, tutorial vignettes, and example data sets.

## Supplementary Material

Reviewer comments

## Data Availability

All validation scripts, reproducibility materials, supplementary tables, and figures are available at https://github.com/NathanBresette/MelsiALL. The Atlas1006 and DietSwap data are available through the R microbiome package (https://microbiome.github.io/). The SKIOME skin microbiome data set is available at NCBI BioProject accession PRJNA554499.

## SUPPLEMENTAL MATERIAL

Supplemental Tables msystems.00407-26-s0001.docx Tables S1 to S5.

OPEN PEER REVIEW reviewer-comments.pdf An accounting of the reviewer comments and feedback.

ASM does not own the copyrights to Supplemental Material that may be linked to, or accessed through, an article. The authors have granted ASM a non-exclusive, world-wide license to publish the Supplemental Material files. Please contact the corresponding author directly for reuse.
