# Supplementary material for: MeLSI: Metric Learning for Statistical Inference in microbiome community composition analysis
Source: mSystems. 2026 Jun 29;11(7):e00407-26. doi: 10.1128/msystems.00407-26 (PMC13386929; doi:10.1128/msystems.00407-26)
Supplement: Supplemental Tables — Tables S1 to S5. [file msystems.00407-26-s0001.docx]

# Supplementary Tables for MeLSI Manuscript

## Supplementary Table S1: Recovery of True Signal Taxa

This table shows MeLSI’s ability to recover true signal taxa using learned feature weights across varying effect sizes and sample sizes.

| Effect Size | Sample Size | n | Precision at 5 | Precision at 10 | Precision at 20 | Recall at 5 | Recall at 10 | Recall at 20 | Mean Rank | AUC-ROC |
| --- | --- | --- | --- | --- | --- | --- | --- | --- | --- | --- |
| Small | 50 | 50 | 0.104 | 0.084 | 0.060 | 0.104 | 0.168 | 0.240 | 50.3 | 0.641 |
| Small | 100 | 50 | 0.104 | 0.084 | 0.064 | 0.104 | 0.168 | 0.256 | 49.3 | 0.649 |
| Small | 200 | 50 | 0.148 | 0.122 | 0.086 | 0.148 | 0.244 | 0.344 | 46.2 | 0.673 |
| Medium | 50 | 50 | 0.356 | 0.262 | 0.197 | 0.178 | 0.262 | 0.394 | 38.9 | 0.733 |
| Medium | 100 | 50 | 0.520 | 0.364 | 0.259 | 0.260 | 0.364 | 0.518 | 31.2 | 0.794 |
| Medium | 200 | 50 | 0.660 | 0.462 | 0.303 | 0.330 | 0.462 | 0.606 | 25.1 | 0.842 |
| Large | 50 | 50 | 0.876 | 0.728 | 0.560 | 0.219 | 0.364 | 0.560 | 26.2 | 0.858 |
| Large | 100 | 50 | 0.976 | 0.912 | 0.705 | 0.244 | 0.456 | 0.705 | 19.7 | 0.914 |
| Large | 200 | 50 | 1.000 | 0.988 | 0.804 | 0.250 | 0.494 | 0.804 | 14.4 | 0.960 |

**Abbreviations:** n, number of simulations; Precision at k, proportion of top-k features that are true signals; Recall at k, proportion of true signals found in top-k features; Mean Rank, average rank of true signal features (lower is better); AUC-ROC, area under receiver operating characteristic curve for classifying signal vs. non-signal taxa based on learned feature weights.

## Supplementary Table S2: Individual Method Comparisons for Power Analysis

This table shows detailed comparisons between MeLSI and each of the five traditional methods individually across all effect sizes and sample sizes. Values are derived directly from simulation output (50 simulations per condition). Note that PERMANOVA F-statistics computed from different distance metrics are not directly comparable because each metric defines a distinct geometric space; F-statistics are reported for within-method assessment.

| Effect Size | n | MeLSI Power (%) | MeLSI F | Euclidean Power (%) | Euclidean F | Bray-Curtis Power (%) | Bray-Curtis F | Jaccard Power (%) | Jaccard F | W. UniFrac Power (%) | W. UniFrac F | UW. UniFrac Power (%) | UW. UniFrac F |
| --- | --- | --- | --- | --- | --- | --- | --- | --- | --- | --- | --- | --- | --- |
| Small | 50 | 6 | 1.230 | 8 | 1.014 | 20 | 1.059 | 0 | 0.987 | 10 | 1.069 | 6 | 1.005 |
| Small | 100 | 10 | 1.342 | 8 | 1.041 | 20 | 1.095 | 6 | 1.012 | 8 | 1.048 | 4 | 1.012 |
| Small | 200 | 16 | 1.432 | 16 | 1.074 | 54 | 1.182 | 6 | 0.988 | 20 | 1.200 | 4 | 0.982 |
| Medium | 50 | 16 | 1.307 | 32 | 1.106 | 74 | 1.325 | 0 | 0.961 | 32 | 1.320 | 2 | 0.954 |
| Medium | 100 | 50 | 1.504 | 52 | 1.207 | 100 | 1.634 | 4 | 1.004 | 82 | 1.705 | 4 | 1.000 |
| Medium | 200 | 96 | 1.780 | 100 | 1.417 | 100 | 2.244 | 0 | 1.005 | 100 | 2.394 | 2 | 0.999 |
| Large | 50 | 84 | 1.585 | 98 | 1.457 | 100 | 2.794 | 10 | 0.976 | 98 | 2.735 | 8 | 0.987 |
| Large | 100 | 100 | 2.129 | 100 | 1.966 | 100 | 4.599 | 4 | 0.987 | 100 | 4.678 | 4 | 1.012 |
| Large | 200 | 100 | 3.129 | 100 | 2.977 | 100 | 8.236 | 6 | 1.002 | 100 | 8.659 | 6 | 1.014 |

**Abbreviations:** Power, empirical statistical power (percentage of simulations with p < 0.05); F, PERMANOVA F-statistic (mean across 50 simulations per condition); Power Difference, MeLSI Power - Traditional Power (%). Results based on 50 simulations per condition. MeLSI used 200 internal permutations; traditional methods used 999 permutations.

**Note:** The five traditional methods are: (1) **Euclidean distance** - standard Euclidean distance on CLR-transformed data; (2) **Bray-Curtis dissimilarity** - count-based dissimilarity metric; (3) **Jaccard dissimilarity** - binary (presence/absence) dissimilarity; (4) **Weighted UniFrac** - phylogenetically-informed distance using abundance-weighted branch lengths; (5) **Unweighted UniFrac** - phylogenetically-informed distance using presence/absence of taxa. Jaccard and Unweighted UniFrac show consistently low power because these presence/absence metrics cannot detect the fold-change effects simulated here (multiplying abundances of already-present taxa does not change their binary presence/absence profiles). F-statistics from different distance metrics should not be directly compared across metrics because each defines a distinct geometric space; they are provided for transparency and within-method assessment across conditions.

## Supplementary Table S3: Individual Method Comparisons for Scalability Analysis

This table shows detailed comparisons between MeLSI and each of the five traditional methods individually across all scalability conditions.

| Condition Type | Condition Value | n | p | Traditional Method | MeLSI Mean F | Traditional Mean F |
| --- | --- | --- | --- | --- | --- | --- |
| **Varying n (p=200)** |  |  |  |  |  |  |
| vary_n | 20 | 20 | 200 | Euclidean | 1.132 | 1.042 |
| vary_n | 20 | 20 | 200 | Bray-Curtis | 1.132 | 1.123 |
| vary_n | 20 | 20 | 200 | Jaccard | 1.132 | 1.031 |
| vary_n | 20 | 20 | 200 | Weighted UniFrac | 1.132 | 1.151 |
| vary_n | 20 | 20 | 200 | Unweighted UniFrac | 1.132 | 1.015 |
| vary_n | 50 | 50 | 200 | Euclidean | 1.277 | 1.085 |
| vary_n | 50 | 50 | 200 | Bray-Curtis | 1.277 | 1.324 |
| vary_n | 50 | 50 | 200 | Jaccard | 1.277 | 0.993 |
| vary_n | 50 | 50 | 200 | Weighted UniFrac | 1.277 | 1.268 |
| vary_n | 50 | 50 | 200 | Unweighted UniFrac | 1.277 | 1.008 |
| vary_n | 100 | 100 | 200 | Euclidean | 1.497 | 1.209 |
| vary_n | 100 | 100 | 200 | Bray-Curtis | 1.497 | 1.660 |
| vary_n | 100 | 100 | 200 | Jaccard | 1.497 | 1.078 |
| vary_n | 100 | 100 | 200 | Weighted UniFrac | 1.497 | 1.684 |
| vary_n | 100 | 100 | 200 | Unweighted UniFrac | 1.497 | 1.064 |
| vary_n | 200 | 200 | 200 | Euclidean | 1.836 | 1.466 |
| vary_n | 200 | 200 | 200 | Bray-Curtis | 1.836 | 2.283 |
| vary_n | 200 | 200 | 200 | Jaccard | 1.836 | 1.026 |
| vary_n | 200 | 200 | 200 | Weighted UniFrac | 1.836 | 2.240 |
| vary_n | 200 | 200 | 200 | Unweighted UniFrac | 1.836 | 1.027 |
| vary_n | 500 | 500 | 200 | Euclidean | 2.511 | 2.008 |
| vary_n | 500 | 500 | 200 | Bray-Curtis | 2.511 | 4.000 |
| vary_n | 500 | 500 | 200 | Jaccard | 2.511 | 1.050 |
| vary_n | 500 | 500 | 200 | Weighted UniFrac | 2.511 | 5.135 |
| vary_n | 500 | 500 | 200 | Unweighted UniFrac | 2.511 | 0.994 |
| **Varying p (n=100)** |  |  |  |  |  |  |
| vary_p | 50 | 100 | 50 | Euclidean | 1.666 | 1.356 |
| vary_p | 50 | 100 | 50 | Bray-Curtis | 1.666 | 2.153 |
| vary_p | 50 | 100 | 50 | Jaccard | 1.666 | 0.964 |
| vary_p | 50 | 100 | 50 | Weighted UniFrac | 1.666 | 1.980 |
| vary_p | 50 | 100 | 50 | Unweighted UniFrac | 1.666 | 1.026 |
| vary_p | 100 | 100 | 100 | Euclidean | 1.670 | 1.333 |
| vary_p | 100 | 100 | 100 | Bray-Curtis | 1.670 | 2.144 |
| vary_p | 100 | 100 | 100 | Jaccard | 1.670 | 0.958 |
| vary_p | 100 | 100 | 100 | Weighted UniFrac | 1.670 | 2.401 |
| vary_p | 100 | 100 | 100 | Unweighted UniFrac | 1.670 | 0.989 |
| vary_p | 200 | 100 | 200 | Euclidean | 1.470 | 1.178 |
| vary_p | 200 | 100 | 200 | Bray-Curtis | 1.470 | 1.614 |
| vary_p | 200 | 100 | 200 | Jaccard | 1.470 | 1.011 |
| vary_p | 200 | 100 | 200 | Weighted UniFrac | 1.470 | 1.676 |
| vary_p | 200 | 100 | 200 | Unweighted UniFrac | 1.470 | 1.063 |
| vary_p | 500 | 100 | 500 | Euclidean | 1.375 | 1.098 |
| vary_p | 500 | 100 | 500 | Bray-Curtis | 1.375 | 1.264 |
| vary_p | 500 | 100 | 500 | Jaccard | 1.375 | 1.028 |
| vary_p | 500 | 100 | 500 | Weighted UniFrac | 1.375 | 1.216 |
| vary_p | 500 | 100 | 500 | Unweighted UniFrac | 1.375 | 1.034 |
| vary_p | 1000 | 100 | 1000 | Euclidean | 1.331 | 1.036 |
| vary_p | 1000 | 100 | 1000 | Bray-Curtis | 1.331 | 1.123 |
| vary_p | 1000 | 100 | 1000 | Jaccard | 1.331 | 1.020 |
| vary_p | 1000 | 100 | 1000 | Weighted UniFrac | 1.331 | 1.147 |
| vary_p | 1000 | 100 | 1000 | Unweighted UniFrac | 1.331 | 1.023 |

**Abbreviations:** n, sample size; p, number of taxa/features; F, PERMANOVA F-statistic (mean across 10 simulations per condition). Results based on 10 simulations per condition.

**Note:** The five traditional methods are: (1) **Euclidean distance** - standard Euclidean distance on CLR-transformed data; (2) **Bray-Curtis dissimilarity** - count-based dissimilarity metric; (3) **Jaccard dissimilarity** - binary (presence/absence) dissimilarity; (4) **Weighted UniFrac** - phylogenetically-informed distance using abundance-weighted branch lengths; (5) **Unweighted UniFrac** - phylogenetically-informed distance using presence/absence of taxa.

## Supplementary Table S4: Parameter Sensitivity Analysis with Standard Deviations

This table shows mean and standard deviation (SD) values for F-statistics, p-values, and computation times across 25 replications per parameter value. These values support the variance analysis discussed in the Parameter Sensitivity section and demonstrate that ensemble learning (B≥10) substantially reduces variance compared to the single-learner baseline (B=1).

| Parameter Type | Parameter Value | n | Mean F (SD) | Mean p-value (SD) | Mean Time (s) (SD) |
| --- | --- | --- | --- | --- | --- |
| **Ensemble Size (B)** |  |  |  |  |  |
|  | 1 | 25 | 1.365 (0.505) | 0.421 (0.290) | 32.9 (1.3) |
|  | 10 | 25 | 1.543 (0.128) | 0.094 (0.175) | 233.0 (4.0) |
|  | 20 | 25 | 1.538 (0.126) | 0.089 (0.155) | 419.8 (6.7) |
|  | 30 | 25 | 1.530 (0.123) | 0.091 (0.156) | 576.8 (6.7) |
|  | 50 | 25 | 1.529 (0.120) | 0.093 (0.165) | 760.0 (11.8) |
|  | 100 | 25 | 1.528 (0.119) | 0.102 (0.165) | 1284.1 (39.8) |
| **Feature Fraction (m_frac)** |  |  |  |  |  |
|  | 0.5 | 25 | 1.578 (0.126) | 0.093 (0.162) | 405.2 (7.0) |
|  | 0.7 | 25 | 1.551 (0.117) | 0.083 (0.155) | 523.7 (8.2) |
|  | 0.8 | 25 | 1.530 (0.123) | 0.091 (0.156) | 578.2 (8.8) |
|  | 0.9 | 25 | 1.517 (0.118) | 0.097 (0.165) | 630.3 (12.7) |
|  | 1.0 | 25 | 1.498 (0.115) | 0.100 (0.159) | 666.7 (11.7) |

**Abbreviations:** B, ensemble size (number of weak learners); m_frac, feature subsampling fraction; n, number of replications; F, PERMANOVA F-statistic; SD, standard deviation; Time, computation time in seconds. Values shown as mean (SD) across 25 replications per parameter value.

**Key findings:** The single-learner baseline (B=1) shows substantially higher variance in F-statistics (SD = 0.505) compared to ensemble approaches (SD = 0.119-0.128 for B≥10), demonstrating that ensemble learning reduces variance and prevents overfitting. F-statistics remained stable across ensemble sizes (B=10-100), with minimal variation in mean values (1.528-1.543) and standard deviations (0.119-0.128).

## Supplementary Table S5: Individual Method Comparisons for Feature Correlation Analysis

This table shows detailed comparisons between MeLSI and each of the five traditional methods individually across all correlation levels.

| Correlation Level | Correlation Value | Traditional Method | MeLSI Power (%) | MeLSI Mean F | Traditional Power (%) | Traditional Mean F | Power Difference (%) |
| --- | --- | --- | --- | --- | --- | --- | --- |
| None | 0.0 | Euclidean | 50.0 | 1.512 | 68.0 | 1.218 | -18.0 |
| None | 0.0 | Bray-Curtis | 50.0 | 1.512 | 100.0 | 1.619 | -50.0 |
| None | 0.0 | Jaccard | 50.0 | 1.512 | 8.0 | 1.024 | 42.0 |
| None | 0.0 | Weighted UniFrac | 50.0 | 1.512 | 64.0 | 1.671 | -14.0 |
| None | 0.0 | Unweighted UniFrac | 50.0 | 1.512 | 6.0 | 1.052 | 44.0 |
| Low | 0.3 | Euclidean | 42.0 | 1.481 | 58.0 | 1.190 | -16.0 |
| Low | 0.3 | Bray-Curtis | 42.0 | 1.481 | 96.0 | 1.500 | -54.0 |
| Low | 0.3 | Jaccard | 42.0 | 1.481 | 6.0 | 0.998 | 36.0 |
| Low | 0.3 | Weighted UniFrac | 42.0 | 1.481 | 40.0 | 1.498 | 2.0 |
| Low | 0.3 | Unweighted UniFrac | 42.0 | 1.481 | 6.0 | 0.992 | 36.0 |
| Moderate | 0.6 | Euclidean | 46.0 | 1.498 | 54.0 | 1.205 | -8.0 |
| Moderate | 0.6 | Bray-Curtis | 46.0 | 1.498 | 100.0 | 1.513 | -54.0 |
| Moderate | 0.6 | Jaccard | 46.0 | 1.498 | 6.0 | 1.000 | 40.0 |
| Moderate | 0.6 | Weighted UniFrac | 46.0 | 1.498 | 32.0 | 1.385 | 14.0 |
| Moderate | 0.6 | Unweighted UniFrac | 46.0 | 1.498 | 10.0 | 0.983 | 36.0 |
| High | 0.8 | Euclidean | 44.0 | 1.507 | 52.0 | 1.208 | -8.0 |
| High | 0.8 | Bray-Curtis | 44.0 | 1.507 | 96.0 | 1.492 | -52.0 |
| High | 0.8 | Jaccard | 44.0 | 1.507 | 6.0 | 1.051 | 38.0 |
| High | 0.8 | Weighted UniFrac | 44.0 | 1.507 | 20.0 | 1.376 | 24.0 |
| High | 0.8 | Unweighted UniFrac | 44.0 | 1.507 | 8.0 | 1.021 | 36.0 |

**Abbreviations:** Power, empirical statistical power (percentage of simulations with p < 0.05); F, PERMANOVA F-statistic (mean across 50 simulations per correlation level); Power Difference, MeLSI Power - Traditional Power (%). Results based on 50 simulations per correlation level.

**Note:** The five traditional methods are: (1) **Euclidean distance** - standard Euclidean distance on CLR-transformed data; (2) **Bray-Curtis dissimilarity** - count-based dissimilarity metric; (3) **Jaccard dissimilarity** - binary (presence/absence) dissimilarity; (4) **Weighted UniFrac** - phylogenetically-informed distance using abundance-weighted branch lengths; (5) **Unweighted UniFrac** - phylogenetically-informed distance using presence/absence of taxa.
