# Supplementary material for: MeLSI: Metric Learning for Statistical Inference in microbiome community composition analysis
Source: mSystems. 2026 Jun 29;11(7):e00407-26. doi: 10.1128/msystems.00407-26 (PMC13386929; doi:10.1128/msystems.00407-26)
Supplement: Reviewer comments [file reviewer-comments.pdf]

AMERICAN SOCIETY FOR MICROBIOLOGY | mSystems®

MeLSI: Metric Learning for Statistical Inference in Microbiome Community Composition Analysis

Nathan Bresette, Aaron Ericsson, Carter Woods, and Ai-Ling Lin

Corresponding Author(s): Ai-Ling Lin, University of Missouri

Review Timeline:

Submission Date:	April 7, 2026
Editorial Decision:	May 19, 2026
Revision Received:	May 26, 2026
Accepted:	June 4, 2026

Editor: Gail Rosen

Reviewer(s): Disclosure of reviewer identity is with reference to reviewer comments included in decision letter(s). The following individuals involved in review of your submission have agreed to reveal their identity: Shi Huang (Reviewer #3)

Transaction Report:

(Note: With the exception of the correction of typographical or spelling errors that could be a source of ambiguity, letters and reports are not edited. The original formatting of letters and referee reports may not be reflected in this compilation.)

DOI: https://doi.org/10.1128/msystems.00407-26

Re: mSystems00407-26 (MeLSI: Metric Learning for Statistical Inference in Microbiome Community Composition Analysis)

Dear Dr. Ai-Ling Lin:

Thank you for the privilege of reviewing your work. Below you will find my comments, instructions from the mSystems editorial office, and the reviewer comments.

Thank you for all your rounds of revisions. If you can consider revising your Table S2 to have the power of each method in the columns, I would greatly appreciate it.

Please return the manuscript within 60 days; if you cannot complete the modification within this time period, please contact me. If you do not wish to modify the manuscript and prefer to submit it to another journal, notify me immediately so that the manuscript may be formally withdrawn from consideration by mSystems.

Revision Guidelines
To submit your modified manuscript, log into the submission site at https://msystems.msubmit.net/cgi-bin/main.plex. Go to Author Tasks and click the appropriate manuscript title to begin. The information you entered when you first submitted the paper will be displayed; update this as necessary. Note the following requirements:

• Upload point-by-point responses to the issues raised by the reviewers in a file named "Response to Reviewers," NOT in your cover letter.
• Upload a compare copy of the manuscript (without figures) as a "Marked-Up Manuscript" file.
• Upload a clean .DOC/.DOCX version of the revised manuscript and remove the previous version.
• Each figure must be uploaded as a separate, editable, high-resolution file (TIFF or EPS preferred), and any multipanel figures must be assembled into one file.
• Any supplemental material intended for posting by ASM should be uploaded with their legends separate from the main manuscript. You can combine all supplemental material into one file (preferred) or split it into a maximum of 10 files with all associated legends included.

For complete guidelines on revision requirements, see our Submission and Review Process webpage. Submission of a paper that does not conform to guidelines may delay acceptance of your manuscript.

Data availability: ASM policy requires that data be available to the public upon online posting of the article, so please verify all links to sequence records, if present, and make sure that each number retrieves the full record of the data. If a new accession number is not linked or a link is broken, provide mSystems production staff with the correct URL for the record. If the accession numbers for new data are not publicly accessible before the expected online posting of the article, publication may be delayed; please contact production staff (mSystems@asmusa.org) immediately with the expected release date.

Publication Fees: For information on publication fees and which article types are subject to charges, visit our website. If your manuscript is accepted for publication and any fees apply, you will be contacted separately about payment during the production process; please follow the instructions in that e-mail. Arrangements for payment must be made before your article is published.

ASM Membership: Corresponding authors may join or renew ASM membership to obtain discounts on publication fees. Need to upgrade your membership level? Please contact Customer Service at Service@asmusa.org.

The ASM Journals program strives for constant improvement in our submission and publication process. Please tell us how we can improve your experience by taking this quick Author Survey.

Thank you for submitting your paper to mSystems.

Sincerely,
Gail Rosen
Editor
mSystems

Reviewer #1 (Comments for the Author):

I appreciate the authors' efforts in addressing all my comments. I have no further comments to add.

Reviewer #2 (Comments for the Author):

Thank you to the authors for answering all my questions. I only have one minor follow-up comment regarding the format of Table S2. It would be better to rearrange it so that each traditional method's power is indicated in columns rather than rows, similar to the format of Table 2. The current format is confusing and might lead readers to mistakenly think all methods reached the same power.

Reviewer #3 (Comments for the Author):

No further comments from me.

Response to Reviewers

Manuscript ID: mSystems00407-26

RESPONSE TO EDITOR

Editor Comment: Thank you for all your rounds of revisions. If you can consider revising your Table S2 to have the power of each method in the columns, I would greatly appreciate it.

Response: We have revised Table S2 as requested. Each method now appears in its own column (empirical power and mean F-statistic), paralleling the format of Table 2. No data values have changed.

Location: Supplementary Table S2 (Supplementary Tables file)

RESPONSE TO REVIEWER #2

Reviewer Comment: Thank you to the authors for answering all my questions. I only have one minor follow-up comment regarding the format of Table S2. It would be better to rearrange it so that each traditional method's power is indicated in columns rather than rows, similar to the format of Table 2. The current format is confusing and might lead readers to mistakenly think all methods reached the same power.

Response: We agree and have rearranged Table S2 accordingly. Each method now appears in its own column, matching the structure of Table 2. No data values have changed.

Location: Supplementary Table S2 (Supplementary Tables file)

Re: mSystems00407-26R1 (MeLSI: Metric Learning for Statistical Inference in Microbiome Community Composition Analysis)

Dear Dr. Ai-Ling Lin:

Your manuscript has been accepted, and I am forwarding it to the ASM production staff for publication. Your paper will first be checked to make sure all elements meet the technical requirements. ASM staff will contact you if anything needs to be revised before copyediting and production can begin. Otherwise, you will be notified when your proofs are ready to be viewed.

Data Availability: ASM policy requires that data be available to the public upon online posting of the article, so please verify all links to sequence records, if present, and make sure that each number retrieves the full record of the data. If a new accession number is not linked or a link is broken, provide production staff with the correct URL for the record. If the accession numbers for new data are not publicly accessible before the expected online posting of the article, publication may be delayed; please contact ASM production staff immediately with the expected release date.

Publication Fees: For information on publication fees and which article types have charges, please visit our website. We have partnered with Copyright Clearance Center (CCC) to collect author charges. If fees apply to your paper, you will receive a message from no-reply@copyright.com with further instructions. For questions related to paying charges through RightsLink, please contact CCC at ASM_Support@copyright.com or toll free at +1-877-622-5543. CCC makes every attempt to respond to all emails within 24 hours.

ASM Membership: Corresponding authors may join or renew ASM membership to obtain discounts on publication fees. Need to upgrade your membership level? Please contact Customer Service at Service@asmusa.org.

Embargo Policy: A press release may be issued as soon as the manuscript is posted on the mSystems Latest Articles webpage. The corresponding author will receive an email with the subject line "ASM Journals Author Services Notification" when the article is available online.

Cover Image Submissions: If you would like to submit a potential Cover Image, please email a file and a short legend to msystems@asmusa.org. Please note that we can only consider images that (i) the authors created or own and (ii) have not been previously published. By submitting, you agree that the image can be used under the same terms as the published article. Image File requirements: TIF/EPS, 7.5 inches wide by 8.25 inches tall (at least 2,250 pixels wide by 2,475 pixels tall), minimum 300 dpi resolution (600 dpi preferred), RGB, and no figure elements, e.g., arrows or panel labels. The legend should be a short description of the image, 1-2 sentences recommended. Please download and use this interactive template in Adobe to ensure that your proposed cover image meets our size requirements (https://journals.asm.org/pb-assets/pdf-text-excel-files/ASM-Interactive-Sizing-Cover-Template-1715689791.pdf).

Thank you for submitting your paper to mSystems.

Sincerely,
Gail Rosen
Editor
mSystems
